# Integrated single-cell and spatial transcriptomics reveal immune landscape and NKT–Th1 signatures in colorectal cancer

**DOI:** 10.3389/fimmu.2026.1774363

**Published:** 2026-06-10

**Authors:** Cunen Wu, Yuanyuan Xu, Ming Zhao, Lihuiping Tao, Dayue Darrel Duan, Jun Qian, Shumin Zhao, Shenlin Liu, Jin-yong Zhou

**Affiliations:** 1 Department of Oncology, Affiliated Hospital of Nanjing University of Chinese Medicine, Jiangsu Province Hospital of Chinese Medicine, Nanjing, Jiangsu, China; 2 Jiangsu Collaborative Innovation Center of Traditional Chinese Medicine Prevention and Treatment of Tumor, Nanjing, Jiangsu, China; 3 Department of Pathology, Affiliated Hospital of Nanjing University of Chinese Medicine, Jiangsu Province Hospital of Chinese Medicine, Nanjing, Jiangsu, China; 4 First Clinical Medical College, Nanjing University of Chinese Medicine, Nanjing, Jiangsu, China; 5 The Academy of Phenomics of TCM, and School of Integrated Medicine, Nanjing University of Chinese Medicine, Nanjing, Jiangsu, China; 6 Department of Pharmacology, University of Nevada Reno School of Medicine, Reno, NV, United States; 7 Jiangsu Province Key Laboratory of Tumor Systems Biology and Chinese Medicine, Affiliated Hospital of Nanjing University of Chinese Medicine, Jiangsu Province Hospital of Chinese Medicine, Nanjing, Jiangsu, China; 8 Department of Key Laboratory, Affiliated Hospital of Nanjing University of Chinese Medicine, Jiangsu Province Hospital of Chinese Medicine, Nanjing, Jiangsu, China

**Keywords:** colorectal cancer, immunotherapy, NKT and Th1 cell related genes, single-cell and spatial transcriptomics, tumor microenvironment

## Abstract

**Background:**

Colorectal cancer (CRC) is a prevalent malignancy with high morbidity and mortality. Immunotherapy benefits only a subset of patients, highlighting the need to explore immune-related signatures associated with response.

**Materials and methods:**

We analyzed a single-cell RNA sequencing dataset of 27,414 cells from tumor core, edge, and matched normal mucosa to investigate NKT cell heterogeneity. A spatial transcriptomics dataset characterized interactions between NKT and Th1 cells in CRC tissues. Integrating nine CRC cohorts from TCGA and GEO, we assessed associations between NKT cell infiltration and clinical outcomes. NKT and Th1 cell–related genes (NTRG) were identified and integrated into an NTRG score, which was systematically validated across multiple independent CRC cohorts to characterize its associations with survival and immunotherapy response. In addition to multi-database validation using several external transcriptomic datasets, we analyzed somatic mutation profiles, GO enrichment, and drug sensitivity patterns associated with different NTRG score groups. Furthermore, key NTRG genes were biologically validated in clinical CRC tissue specimens using immunohistochemistry.

**Results:**

Elevated NKT cell infiltration correlated with improved prognosis and enhanced immunotherapy sensitivity. Spatial analysis revealed NKT and Th1 co-localization mediated by COLLAGEN signaling. The NTRG score quantified tumor–immune properties, and high scores were linked with worse overall survival yet stronger immunotherapy response. Validation confirmed differential NTRG expression between normal and cancerous tissues and across immunotherapy responders and non-responders.

**Conclusion:**

NTRG is an exploratory multi-gene signature associated with the immune landscape of colorectal cancer, offering a foundation for future investigation into its relationship with immunotherapy response and tumor microenvironmental organization.

## Graphical Abstract

**Figure:**
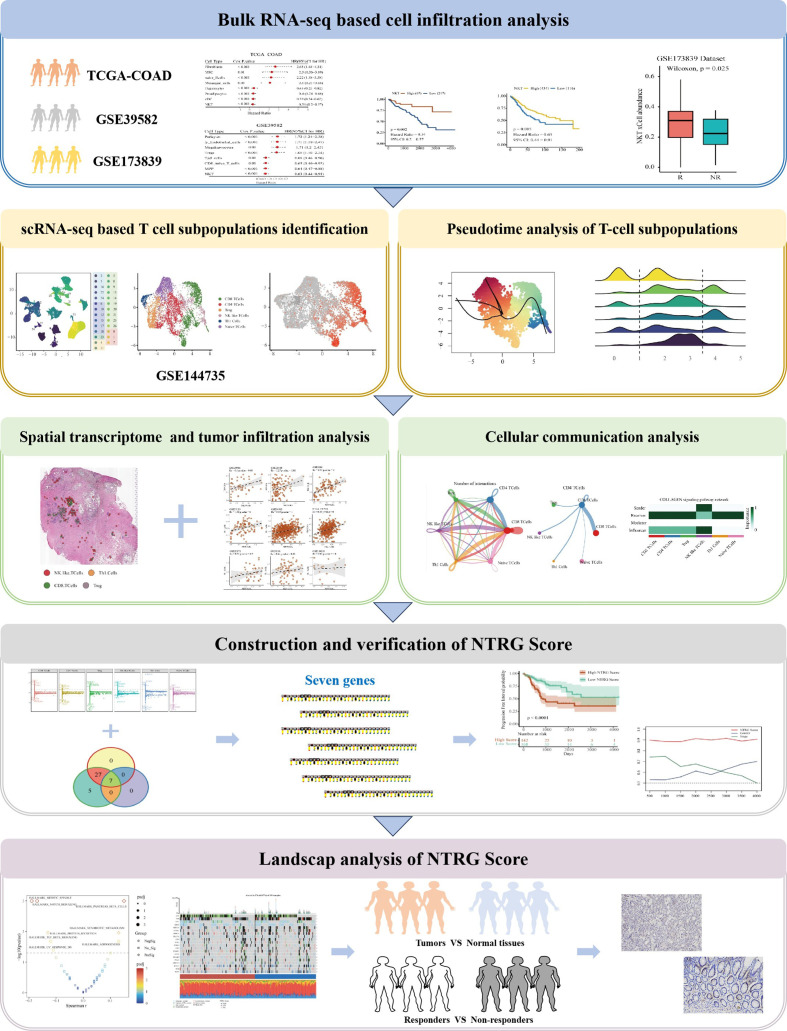
We obtained datasets from TCGA and NCBI, explored NKT cell heterogeneity at the single-cell level, and developed a risk model to predict patient outcomes. We performed GSVA, somatic mutation, GO enrichment, and drug sensitivity analyses, and validated key molecular expressions in clinical samples, aiming to provide new insights for colorectal cancer immunotherapy.

## Introduction

Colorectal cancer (CRC) has been threatening human health as one of the most common digestive system malignancies. According to the survey from the International Agency for Research on Cancer (IARC), 1.9 million of new cases and 935,000 fatalities due to CRC have been confirmed in 2020 (1). Even worse, the incidence and mortality of CRC are increasing on account of changes in diet and an aging population in China (2). Despite of remarkable treatment achievements made in recent decades, limited therapeutic strategies and curative effect are still restricting the prognosis of patients who progress after standard therapy (3). Immune checkpoint inhibitors (ICIs) have presented considerable efficacy in specific population of CRC characterized as microsatellite instability-high (MSI-H) or mismatch repair deficiency (dMMR). Notably, the proportion of the sensitive groups only accounts for less than 5% of the whole cases. Inversely, the degree of benefit from ICIs of the vast majority of patients in a state of microsatellite stable (MSS) and mismatch repair proficiency (pMMR) varies widely and is quite difficult to be predicted exactly (4). Hence, there is an urgent need to identify immune-related signatures that are associated with immunotherapy efficacy in CRC.

Natural killer T (NKT) cells are a special subset of T lymphocytes that can express both T-cell and NK cell receptors and recognize the lipid antigens presented by non-classical major histocompatibility complex (MHC) molecule CD1d (5). It has been verified that NKT cells play an immuno-surveillance role, as the decreased level of which induced by epigenetic modifications results in higher incidence and pathologic severity in CRC (6). Stimulation of NKT cells is capable of reinvigorating exhausted CD8 T cells to sensitize CRC to anti-PD-1 treatment (7). Moreover, higher infiltration of NKT cells has been authenticated to be an independent factor for favorable prognosis including extended overall and disease-free survival rates in CRC (8). Diminished antitumor immunity originated from lessened accumulation of NKT cells together with limited T helper 1 (Th1) function has been found to promote CRC progression (9). Additionally, Th1-biased immune response stemming from NKT cell-mediated cytokine profiles has shown vital implications in potential anticancer and immunostimulating therapy (10). Therefore, the malignant features of CRC depend to some extent on the interaction of NKT and Th1 cells, deep research on which may provide insight into the pathogenic and prognostic process.

Single-cell sequencing technology is characterized by identifying the diversity between cell types to reveal cellular variations in different states (11). The spatial transcriptome combines histological imaging and RNA sequencing to quantitatively detect gene expression while providing corresponding spatial location information (12). Hence, integrated single-cell and spatial transcriptomic profiling is giving rise to the access to tumor heterogeneity identification, in order for promising biomarkers as well as more precise tumor immunotherapy (13).

During our present research, CRC-related datasets were obtained from The Cancer Genome Atlas (TCGA) and Gene Expression Omnibus (GEO) databases. The heterogeneity of NKT cells at the single-cell level as well as its interaction with Th1 cells were mainly studied. An NTRG score model was then constructed to characterize its associations with prognosis and immunotherapy response in CRC, based on which immune cell type scores and treatment-response patterns were further evaluated. Analyses on gene set variation, somatic mutation landscape, immune cell infiltration, gene enrichment, drug sensitivity, and cell interaction were investigated successively. Additionally, paired CRC samples were collected to explore the expression difference of NTRG. Our research aimed to gain molecular insight into the mechanism underlying the immune microenvironment of CRC, which may pave way for more precise immunotherapy strategies and even novel therapeutic targets to benefit more patients. Our research is compliant with the TITAN Guidelines 2025 (14).

## Materials and methods

### Data download

Log2(count+1)-processed bulk RNA-seq data, MuTect2-processed somatic mutation data, and corresponding clinical information of 462 colorectal adenocarcinoma (COAD) patients from TCGA were obtained through the UCSC Xena site (https://xenabrowser.net/datapages/). RNA-seq data and sample annotation information including binary best overall response (BOR), tumor mutation burden (TMB), tumor neoantigen burden (TNB), and survival information of the anti-PD-L1 therapy cohort (IMvigor210, n=298) were obtained using the IMvigor210CoreBiologies R package (version 1.0.0) (15). Datasets including GSE144735 (the KU Leuven dataset containing a total of 27,414 cells in the core and edge regions of tumor, together with matched normal mucosal tissues from six Belgian CRC patients), GSE173839, GSE39582, GSE20916, GSE21510, GSE5206, GSE33113, GSE23878, GSE9348, and GSE110224 were downloaded from the GEO database of the National Center for Biotechnology Information (NCBI). The clinical data and survival data were then organized (**Supplementary Table 1**).

Spatial transcriptome (ST) data including h5ad file and the original image of CRC were acquired from the study of Wu et al. (16). ST data were processed and analyzed through Python package Scanpy (v.1.9.1). Spatial coordinates with a total number greater than 20,000, the number of expressed genes greater than 6,000, and the proportion of mitochondrial genes greater than 10% were filtered. The “Normalize_total” function was used to normalize the count and spatial information data, and the “highly_variable_genes” function was applied to extract the top 2,000 highly variable genes. Then, principal component analysis (PCA), uniform manifold approximation and projection (UMAP), and Leiden algorithm were used for dimensionality reduction and cluster analysis.

### Raw data processing and quality control for single-cell sequencing

Cell Ranger (version 2.2.0) was used to process raw data, demultiplex cell barcodes, map reads to transcriptome, and downsample reads (generate normalized aggregated data across samples as needed). A raw unique molecular identifier (UMI) count matrix was produced and then converted into Seurat objects via R package Seurat (version 4.0.6). Cells with a UMI count less than 500 or a mitochondrially derived UMI count more than 20% were considered low quality and removed. To eliminate potential dual genes, single cells in which more than 6,000 genes were detected were also filtered out, and the remaining cells were applied for downstream analysis.

The UMI count matrix was log-normalized after quality control. Patient numbers were used to eliminate potential batching effects as samples from six patients were processed and sequenced in batches. The first 2,000 variable genes were selected to create potential Anchors that functioned as Seurat FindIntegration Anchors. Subsequently, the data were integrated using the IntegrateData function to create a new matrix with 2,000 features, in which the potential batch effects were regressed. To reduce the dimensionality of the scRNA-Seq dataset, PCA was performed on the integrated data matrix. The first 20 PCs were chosen for downstream analysis using the Elbowplot function of Seurat. The FindClusters function provided by Seurat with the resolution set to the default (res=0.8) was applied to identify the main cell clusters, which was then visualized with the UMAP plot. Conventional markers described in previous studies were used to classify each cell into a known biological cell type. The FindAllMarkers function of the Seurat package was used to identify cell clusters and differentially expressed genes between cell types. All single-cell data analysis and integration were implemented by R software Seurat (version 4.0.6). Two-cell quality control was implemented using the R Scrublet package. The cells with less than 300 genes in a single cell were deleted by quality control. Moreover, cells with more than 20% mitochondrial gene reads were deleted. The data normalization and standardization of each sample were achieved by PCA, and the between-batch difference between samples was adjusted by the Harmony package. The dimensionality reduction and visualized of single-cell data were realized by the UMAP algorithm. Genes with log-fold change (logFC) ≥1 and false discovery rate (FDR)<0.05 were considered as specifically upregulated genes.

### Evaluation of clinical efficacy

The primary outcomes were objective response rate (ORR) and overall survival (OS). ORR was assessed with the Response Evaluation Criteria in Solid Tumors (RECIST, version 1.1) in all cohorts except Hugo 2016, in which ORR was evaluated with immune-related RECIST (irRECIST). Patients were divided into two groups according to their response status. Complete response (CR) and partial response (PR) were defined as responders (R, response), stable disease (SD), and progressive disease (PD) were recognized as non-responders (NR, non-response).

### Gene set variation analysis

The “h.all.v7.5.2.symbols” gene set was obtained from the MSigDB database, and the gene set variation analysis (GSVA) was performed on the characteristic genes of NKT and Th1 cells in the single-cell dataset (the GSVA analysis parameters were selected as the default settings). The scores of NKT and Th1 cell-related genes in TCGA pan-cancer dataset were calculated. Patients were divided into high- and low-risk groups by ROC extraction of the best cutoff value, and the enrichment of various pathways was further explored. Wilcoxon analysis was proceeded to explore the relationship between NKT and Th1 cell-associated gene scores and the efficacy of immunotherapy.

### Construction of the NTRG score

The characterized genes of NKT and Th1 cells were screened by R package Seurat. Univariate Cox analysis was performed on these characterized genes in TCGA-COAD dataset to screen important genes associated with OS. The annotations were then analyzed by Gene Ontology (GO).

In addition, important genes related to immunotherapy efficacy in the IMvigor210 and GSE173839 datasets were screened. The intersection was taken in these screened gene sets to obtain CRC prognosis and immunotherapy efficacy-related genes.

The NTRG score model was constructed based on these CRC prognosis and immunotherapy efficacy-related genes, which was designed to assess the impact on OS and immunotherapy efficacy by integrating the expression levels of genes characterized by NKT and Th1 cells, and the specific construction of the score formula was as follows:


$$NTRG\;Score=\sum_{i}Coef(gene\;i)*Exp(gene\;i)$$

The coefficients were generated using a multivariate Cox proportional hazards model in the TCGA-COAD training cohort. The final model was determined via stepwise regression, using the expression levels of the seven genes as covariates and overall survival as the endpoint.

### Somatic mutation analysis

Somatic mutation information from TCGA-COAD samples was downloaded in “MAF” format. Waterfall plots were subsequently generated using the “Maftools” program in R software in order to visualize and summarize all mutated genes.

### Immune cell infiltration assessment and tumor purity analysis

An immune-infiltrating cell score was generated for each sample based on the expression profile using XCell and Estimate analysis via R package IOBR, a computational tool commonly applied in the research of immuno-tumor biology.

### SPOTlight analysis

SPOTlight deconvolution (version 1.0.0) was performed on the spatial transcriptome time course by integrating scRNA-seq cell type profiles to determine spatial interactions. The single-cell reference dataset was first converted into a SingleCellExperiment object and log-normalized using scater::logNormCounts. Gene variance was calculated using scran::modelGeneVar. After excluding ribosomal protein genes (^Rp[l|s]) and mitochondrial genes (^Mt), the top 3,000 highly variable genes were selected. Marker genes for each cell type were identified using scran::scoreMarkers. Genes with a mean AUC >0.4 were retained and sorted in descending order of their AUC values. To balance the computational load, a maximum of 100 cells were randomly downsampled per cell type. With the processed single-cell data as the reference and the spatial transcriptomics data (Seurat object) as the target, the SPOTlight function was executed. The parameters were configured as follows: groups = cell type annotations, mgs = marker gene dataframe, hvg = highly variable genes, weight_id = “mean.AUC,” group_id = “cluster,” gene_id = “gene”. All other parameters were kept at their default settings (model = “ns,” min_prop = 0.01). This generated a cell type probability matrix (mat) for each spatial spot, which was subsequently used for spatial cell type mapping. This analytical pipeline was applied independently for both the global cell type annotation and the fine-grained T-cell subpopulation annotation.

### Gene enrichment analysis

R package ClusterProfiler (version 4.6.0) was used to perform GO function annotation on all differentially expressed genes to identify significantly enriched biological processes covering biological process (BP), molecular function (MF), and cellular component (CC), which were visualized by R package GOplot (version 1.0.2), and the significance threshold was set as p<0.05.

### Genomics of drug sensitivity in cancer analysis

Genomics of drug sensitivity in cancer (GDSC) study was implemented based on the largest publicly pharmacogenomics database GDSC (https://www.cancerrxgene.org/). The prediction process was finished through R package pRRophetic (version 0.5). The half-maximal inhibitory concentration (IC50) was estimated by ridge regression, and the accuracy was evaluated by 10-fold cross-validation based on the GDSC training set. All parameters were set by default values. The batch effect of “Combat” and the tissue type of “allSoldTumurs” were removed, and duplicate gene expression was summarized as the mean value.

### Cell interaction analysis

The CellChat (version1.1.3) R package was used to quantitatively infer and analyze the intercellular communication network from single-cell RNA sequencing data. The differences in cell interaction between CRC and normal colon tissue samples were compared. Then, a circle diagram was used to show the cell interaction of cell subsets in CRC tissue at the single-cell level. A bubble plot was selected to analyze all important receptor and ligand pairs in cell–cell signal transduction.

### Pseudotime analysis

The slingshot algorithm (version 2.18.0) was employed to infer the developmental trajectories of CRC and associated immune cells. PCA was performed on all cells to preserve major biological variation between cell states. Slingshot was then operated on the first three major principal components, with the initial state as the starting cluster. Using the Slingshot routine analysis flow, pseudotime values and assigned cell type branches were obtained. Ambiguous cells from the corresponding branches that were not part of the enriched state were removed to ensure the accuracy of the trajectories. Single-cell pseudotime traces were analyzed by newCellDataSet(), estimateSizeFactors(), and estimateDispersions() in R package Monocle2 (version 2.26.0). Cells with low-quality were filtered out via detectGenes() with the parameter “min_expr = 0.1”.

### Immunohistochemical staining

Paraffin sections were incubated in xylene and dehydrated in pure ethanol, followed by dehydration in gradient ethanol. Slides were immersed in sodium citrate antigen retrieval solution. Sections were immersed in 3% H_2_O_2_ and incubated at room temperature away from light. Objective tissues were covered with 3% BSA at room temperature for 30 min. Slides were incubated with primary antibody overnight at 4 °C. After washing with PBS, objective tissues were covered with secondary antibody labeled with HRP and incubated at room temperature for 50 min. DAB chromogenic reagent was added, and reaction time was managed by observing in microscopy until the nucleus shows brown yellow. Counterstain in nucleus was performed using hematoxylin staining solution. Slides were dehydrated successively in gradient ethanol, pure ethanol, and xylene, followed by mounting with resin mounting medium. For result interpretation, nuclei were shown in blue and the positive cells were characterized as brown yellow.

The inclusion criteria contains the following: (1) pathological diagnosis meets the diagnostic criteria for colorectal adenocarcinoma/mucinous adenocarcinoma; (2) pathologically confirmed stage II/III and has implemented radical surgery; (3) patients are volunteers and have signed informed consent. Clinical information is shown in **Supplementary Table 2**. The protein expression levels in the immunohistochemically (IHC) stained sections were semiquantitatively evaluated using the average optical density (AOD) method. The AOD value was calculated using the formula: AOD = integrated density (IOD)/positive area.

### Statistical analysis

Data processing and bioinformatic analysis were performed via R software (version 4.1.3). For comparisons of continuous variables between two groups, statistical significance of normally distributed variables was estimated by independent Student t-test, and differences between those that were not normally distributed were analyzed by Mann–Whitney U test (Wilcoxon rank-sum test). The chi-square or Fisher’s exact test was applied to compare the statistical significance between the two groups of categorical variables. The Survival R package was used to perform survival analysis. Kaplan–Meier (KM) survival curves were conducted to show survival differences. Log-rank test was employed to assess the differences in survival time. Univariate and multivariate Cox analyses were based on the survival R package, and p<0.05 was judged as significantly different.

## Results

### NKT cells correlate with survival and response to immunotherapy

**Figure 1:**
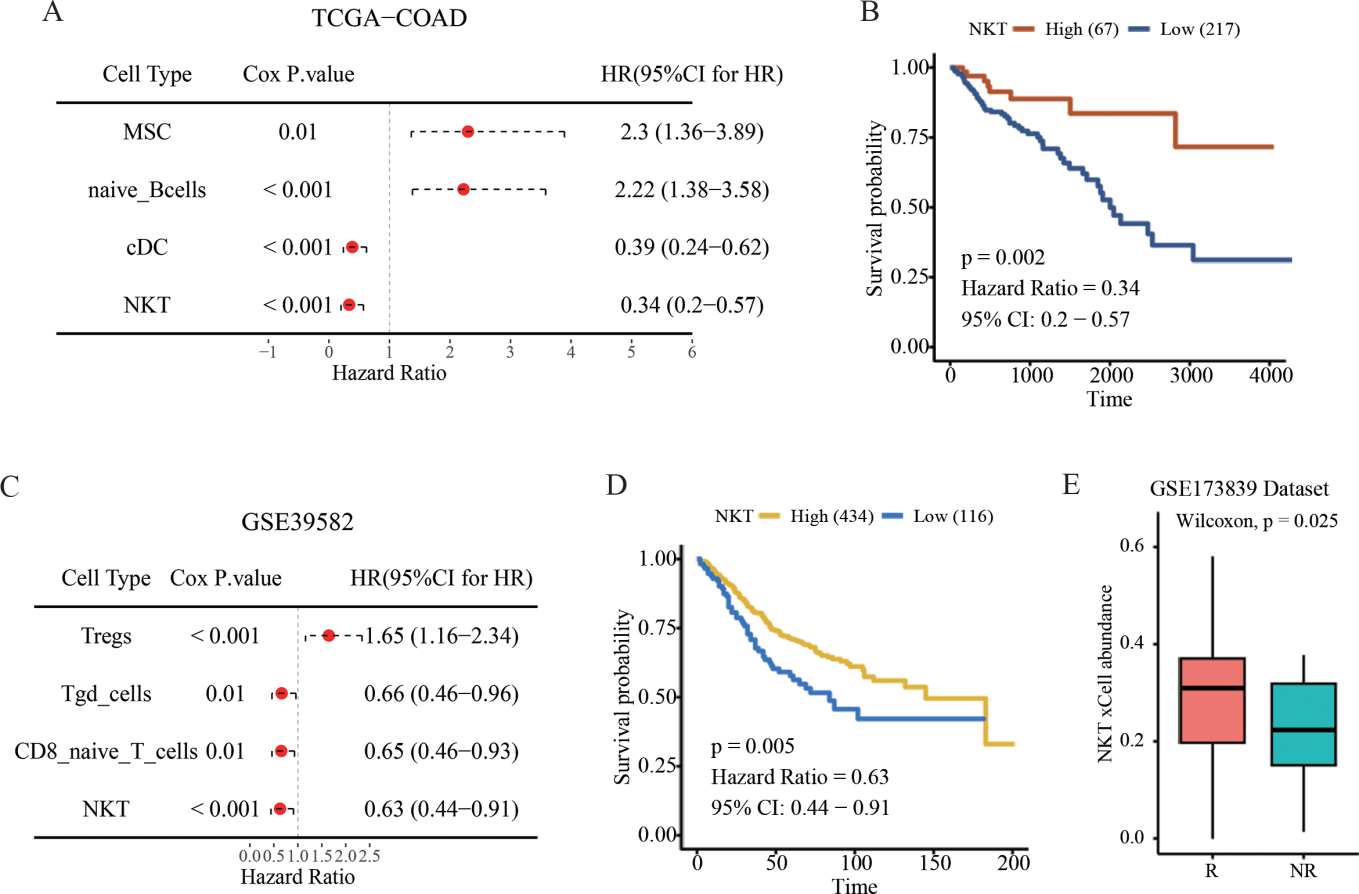
Correlation between NKT cell infiltration and prognosis. **(A)** Relationship between immune cell infiltration and OS in TCGA-COAD dataset. **(B)** Relationship between NKT cell infiltration and prognosis verified by the Kaplan–Meier curve in TCGA-COAD dataset. **(C)** Relationship between immune cell infiltration and OS in the GSE39582 dataset. **(D)** Relationship between NKT cell infiltration and prognosis verified by Kaplan–Meier curve in the GSE39582 dataset. **(E)** Role of NKT cell infiltration in immunotherapy response in the GSE173839 dataset, R stands for Responders and NR stands for Non-responders.

Inferential analysis of cellular infiltration based on bulk RNA-seq data from TCGA-COAD indicated that four immune cell types are associated with OS significantly in the univariate Cox regression model (**Figure 1A**). Among them, high infiltration of MSC and naive B cells contributed to worse OS, whereas that of classical dendritic and NKT cells made for better OS. Furthermore, XCell analysis was performed in the GSE39582 dataset to evaluate the infiltration status of immune cells and its association with OS. Univariate survival analysis revealed that Treg cells lead to poor prognosis. Nevertheless, high infiltration of γdertaT, naive CD8T cells, and NKT cells resulted in better prognosis (**Figure 1C**). Notably, patients with higher NKT cell infiltration presented more favorable prognosis in both data sets (**Figures 1B, D**). Additionally, in the GSE173839 cohort that received devarumab treatment, the abundance of NKT cells was found to be relatively higher among those who responded to immunotherapy positively (**Figure 1E**).

### Single-cell characteristics of colorectal cancer samples

**Figure 2:**
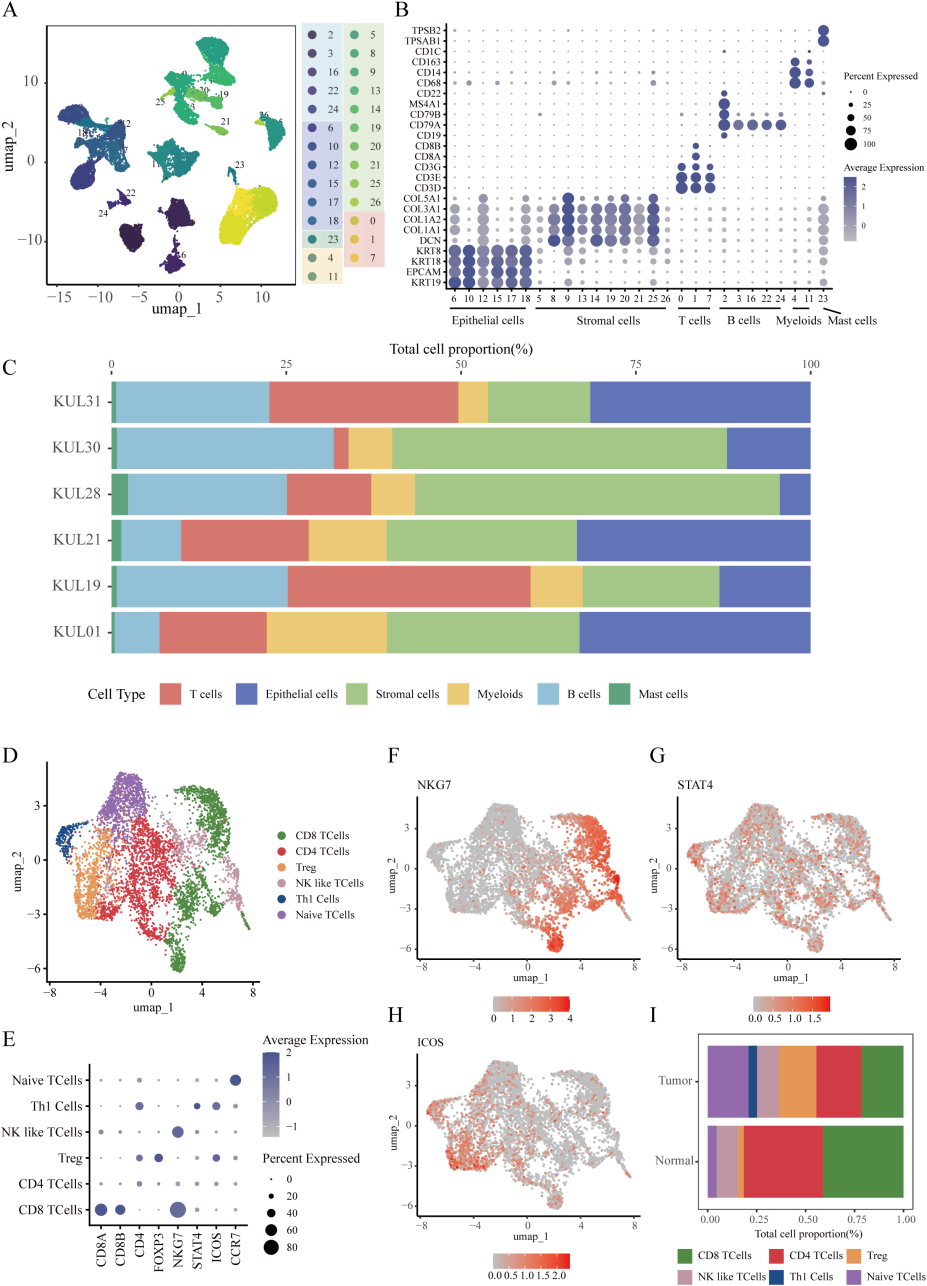
Identification of T-cell subpopulations by single-cell analysis. **(A)** Single-cell UMAP map of CRC. **(B)** Expression of marker genes in different cell subtypes of CRC. **(C)** Proportion of cell subsets in different samples. **(D)** UMAP of T cells in CRC. **(E)** Expression of marker genes in T-cell subtypes in CRC. **(F)** NKG7 expression distribution in the single-cell dataset. **(G)** STAT4 expression distribution in the single-cell dataset. **(H)** ICOS expression distribution in the single-cell dataset. **(I)** Distribution of T-cell subsets in tumor and normal samples.

To further explore the heterogeneity of NKT cells at the single-cell level, we included a scRNA-seq dataset consisting of six CRC samples. A total of 27,414 cells were obtained after quality control, and 26 cell subclasses were identified through the R package Seurat (**Figure 2A**). The cell subclasses were annotated by cell markers and divided into six cell subtypes, namely, 6,168 epithelial cells (KRT19, EPCAM, KRT18, and KRT8), 7650 stromal cells (DCN, COL1A1, COL1A2, COL3A1, and COL5A1), 2,676 myeloid cells (CD68, CD14, CD163, and CD1C), 5,770 T cells (CD3D and CD3E), 4,902 B cells (CD79A and CD19), and 248 mast cells (TPSAB1 and TPSB2). The expression of all cell marker genes is shown in **Figure 2B**. The proportion of the six cell types in each patient was then analyzed. The results showed that the distribution of each cell type in all patients is relatively uniform, suggesting reliable sequencing quality and high consistency. In addition, a higher percentage of stromal cells and epithelial cells were detected in all the six samples (**Figure 2C**). Next, 5,770 T cells were separated into six subsets containing 1,627 CD8 T cells (CD8A and CD8B), 1,572 CD4 T cells (CD4), 790 Treg cells (FOXP3), 551 NKT cells (CD8A+ and NKG7+), 223 Th1 cells (STAT4 and ICOS), and 1,007 naive T cells (CCR7) (**Figure 2D**). The expression of relative marker genes for all T-cell subsets is presented in **Figure 2E**. The distribution of NKG7, STAT4, and ICOS gene expression in T-cell subsets was exhibited (**Figures 2F–H**). The results revealed that the content of CD4T and CD8T cells in normal samples is higher, whereas that of Naive T, Treg, and Th1 cells increased apparently in CRC tissues (**Figure 2I**).

### T-cell-related pseudotime analysis

**Figure 3:**
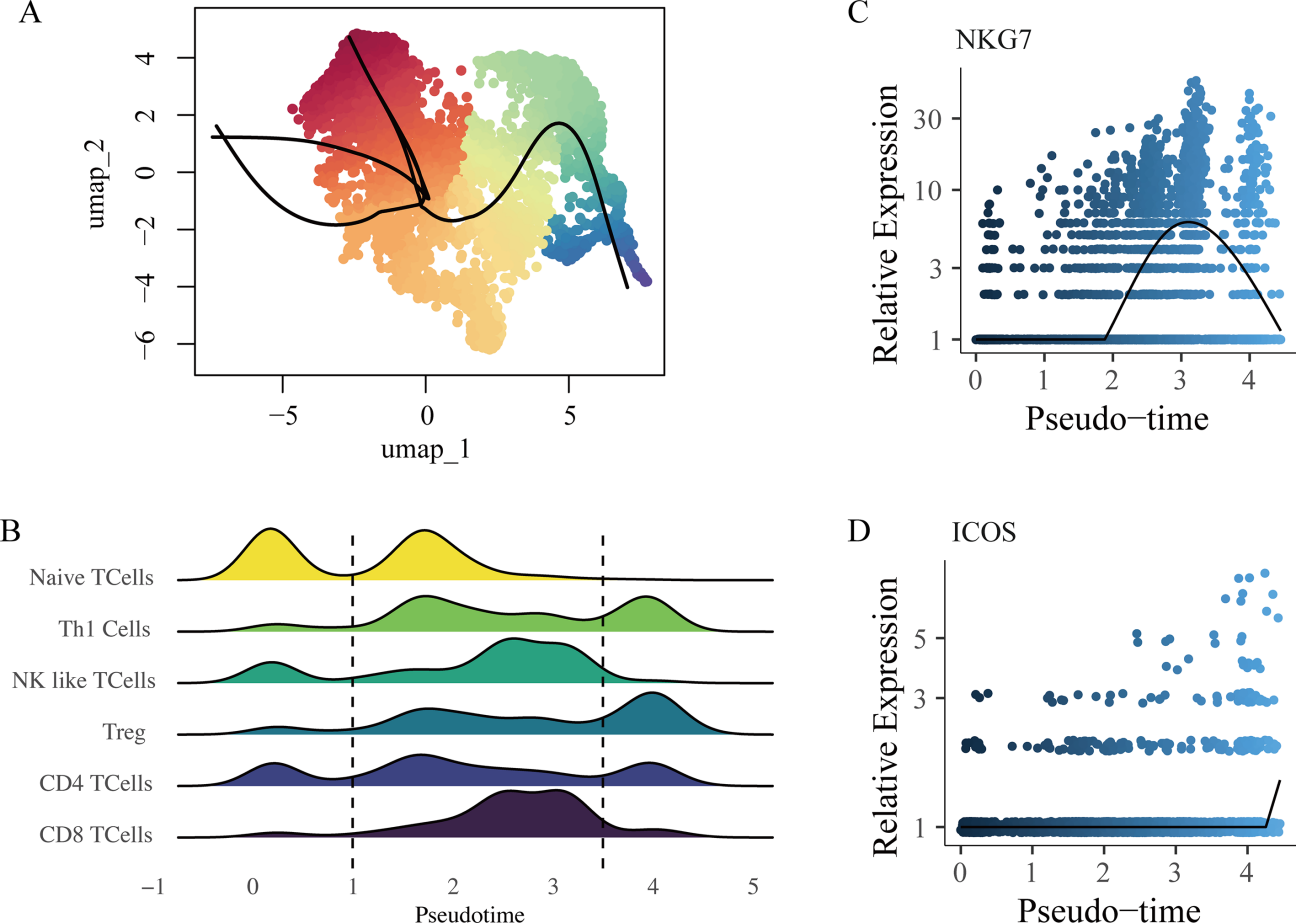
Pseudotime analysis of T-cell subpopulations. **(A)** Slingshot analysis of T-cell subsets. **(B)** Monocle2 analysis of the distribution of T-cell subsets. **(C)** Pseudotime distribution of NKG7 gene expression. **(D)** Pseudotime distribution of ICOS gene expression.

The T-cell subsets were analyzed to explore their potential differentiation trajectories. As shown in the pseudotime ordering, T-cell subsets exhibited a continuous transcriptomic state transition originating from naive T cells and branching toward three distinct terminal states: Th1, Treg, and NKT cells (**Figure 3A**). The distribution of T-cell subsets along these inferred trajectories suggested that the developmental progression potentially begins with naive T cells, traversing through intermediate CD8+ and CD4+ T-cell states. Along this pseudotime axis, NKT cells emerged at an intermediate-to-late stage, whereas Th1 and Treg cells predominantly mapped to the terminal ends of the trajectories (**Figure 3B**).

Furthermore, plotting gene expression along pseudotime revealed that the expression level of NKG7, a marker gene of NKT cells, was low at the beginning of the trajectory, increased gradually as cells transitioned toward intermediate states, and then decreased slightly at the terminal ends (**Figure 3C**). In contrast, the expression of ICOS, a marker associated with Th1 cells, was significantly elevated only at the terminal stage of the inferred trajectory (**Figure 3D**). The expression trends of these signature genes align with the inferred cellular ordering, suggesting a stepwise maturation and functional specialization among different T-cell subsets within the tumor microenvironment.

### Relationship between NKT and Th1 cells in spatial location and tumor infiltration

**Figure 4:**
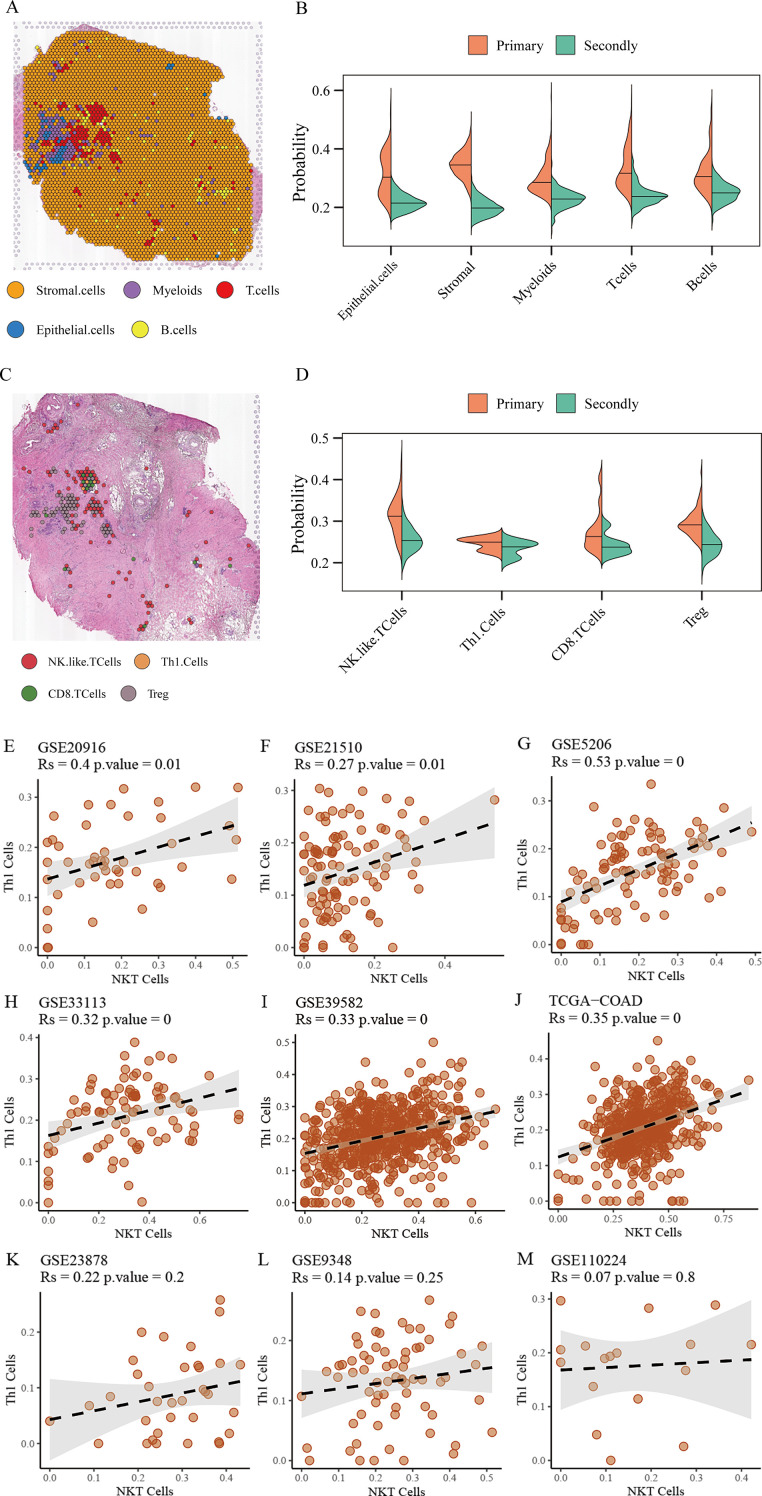
Analysis on cell subpopulation by spatial transcriptome and tumor infiltration. **(A)** Spatial location annotation of different cells. **(B)** SPOTlight cell annotation analysis. **(C)** Annotation of the spatial location of T-cell subsets. **(D)** SPOTlight cell annotation analysis of T-cell subsets. **(E–M)** Scatter plot of the correlation between NKT and Th1 cell infiltration in the GSE20916 **(E)**, GSE21510 **(F)**, GSE5206 **(G)**, GSE33113 **(H)**, GSE39582 **(I)**, TCGA-COAD **(J)**, GSE23878 **(K)**, GSE9348 **(L)**, and GSE110224 **(M)** datasets.

One CRC spatial transcriptome sequencing sample was analyzed to further characterize the relationship between NKT and other T cells. Firstly, the cell types in the spatial location were annotated by the Spotlight algorithm (**Figure 4A**). The results showed that most of the areas in the sample were stromal cells, and T cells were mainly distributed around epithelial cells. **Figure 4B** presents the likelihood of each cell type in the Spot. Next, the spatial location of T-cell subsets was further annotated. A total of four different T-cell subsets were annotated, namely, NKT, Th1, CD8T, and Treg cells. Among them, NKT and Th1 cells were discovered to be co-localized in space, between which a certain regulatory relationship might exist (**Figure 4C**). **Figure 4D** exhibits the possibility of cell subsets of Spot in the annotation of T-cell subsets, implying a credible spatial annotation effect. Nine public datasets of CRC were recruited in order for further verification of the underlying correlation between NKT and Th1 cells. The characteristic genes of NKT and Th1 cells were scored by ssGSEA. The results of Spearman correlation analysis showed that there is a positive correlation between the infiltration degree of NKT and Th1 cells in six datasets containing GSE20916, GSE21510, GSE5206, GSE33113, GSE39582, and TCGA-COAD (**Figures 4E–J**). The correlation in other three datasets (GSE23878, GSE9348, and GSE110224) presented a certain positive trend although without statistical significance (**Figures 4K–M**).

### Cell interaction analysis of NKT and Th1 cells

**Figure 5:**
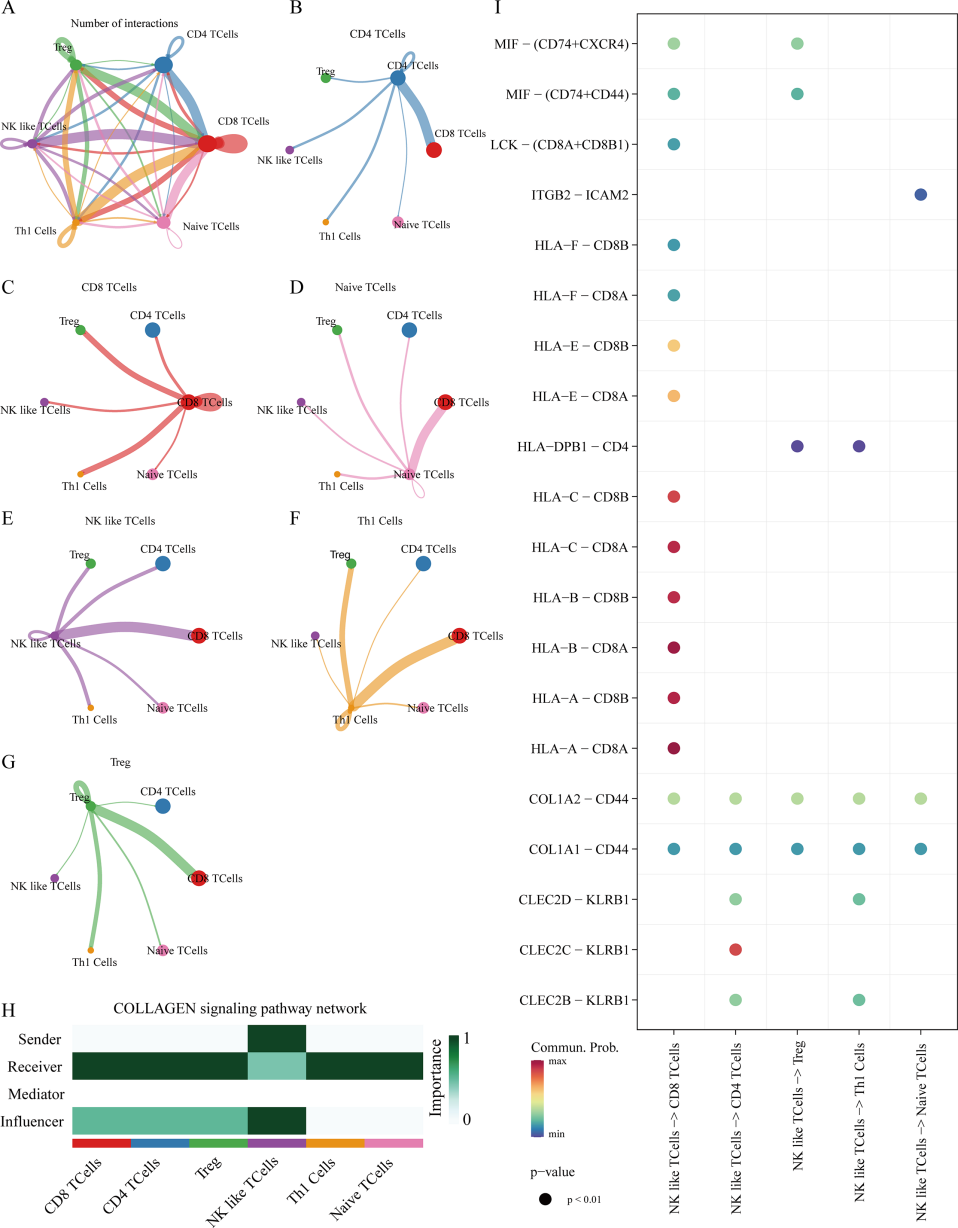
Cellular communication analysis between T-cell subsets. **(A)** Number of interactions of cell communication between various cell types. **(B–G)** Number of communication interactions in CD4T **(B)**, CD8T **(C)**, naive T **(D)**, NKT **(E)**, Th1 **(F)**, and Treg cells **(G)**. **(H)** Ability of different cell subsets to receive or emit signals in the COLLAGEN signaling pathway. **(I)** Receptor and ligand pairs for NKT cells to interact with other T-cell subsets.

CellChat was employed to infer and quantify the communication relationship between six types of T cells, and the number of cell communication was visualized in a circle chart (**Figure 5A**). The numbers of exchange interactions between CD4T, CD8T, naive T, NKT, Th1, Treg, and other cells are exhibited in **Figures 5B–G**. Of note, CellChat analysis indicated that NKT cells possess the strongest ability to receive cellular communication signals from other cells through the COLLAGEN pathway (**Figure 5H**). Furthermore, it was found that COL1A2, the receptor of NKT cell subsets, is capable of binding to the ligand of Th1 cell CD44 to realize communication between the two cell types via activating COLLAGEN signaling (**Figure 5I**).

### Construction and validation of the NTRG score

**Figure 6:**
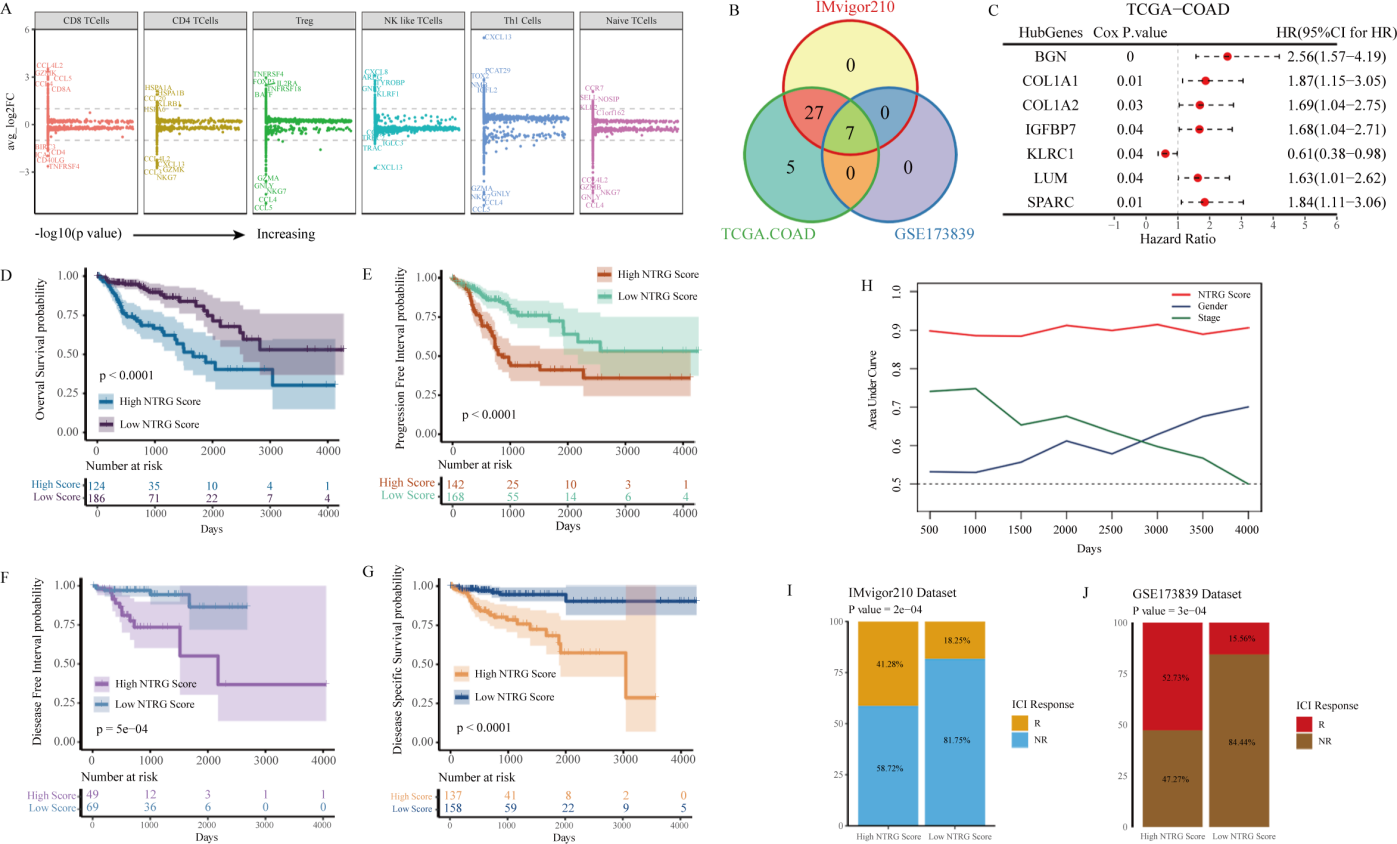
Establishment and verification of NTRG score as a prognostic predictor. **(A)** Feature gene screening of T-cell subsets based on single-cell datasets. **(B)** Intersection of prognosis and immune response-related genes from TCGA-COAD, IMvigor210, and GSE173839 datasets. **(C)** Relationship between seven important genes and OS. **(D–G)** Relationship between NTRG score and OS **(D)**, PFI **(E)**, DFI **(F)**, and DSS **(G)**. **(H)** Performance analysis of NTGR score in predicting OS of patients in TCGA-COAD dataset. **(I)** Validation of the relationship between the NTGR score and immunotherapy response in the IMvigor210 dataset. **(J)** Research on the linking between NTGR score and immunotherapy efficacy in the GSE173839 dataset.


Characteristic genes of NKT and Th1 cells were screened through R package Seurat (**Figure 6A**). Then, univariate Cox analysis was performed on the above NKT and Th1 cell signature genes in the TCGA-COAD dataset to find out important genes associated with OS, whose biological functions were then annotated via Go analysis (**Supplementary Figure 1**). Based on the above genes, fundamental genes relevant to immunotherapy efficacy were further filtrated in IMvigor210 and GSE173839 datasets. Seven closely related genes covering BGN, COL1A1, COL1A2, IGFBP7, KLRC1, LUM, and SPARC were eventually obtained through intersection of the three gene sets (**Figure 6B**). The relationship between these seven genes and OS in TCGA-COAD dataset is shown in **Figure 6C**. The NTRG Score was constructed based on these seven important genes, and the specific scoring formula was as follows:

$$\text{NTRG score}=\;0.0023059979\times Exp_{BGN}-0.0013929950\;\times Exp_{COL1A1}+0.0012700509\times Exp_{COL1A2}-0.0004943891\;\times Exp_{IGFBP7}+0.1728978045\;\times Exp_{KLRC1}-0.0028620101\;\times Exp_{LUM}+0.0017296451\;\times Exp_{SPARC}$$

The correlation between NTRG score and prognosis was verified in TCGA-COAD dataset. It was presented that patients with a higher NTRG score had worse OS, PFI, DFI, and DSS (**Figures 6D–G**). The association between NTRG score and prognosis of patients was evaluated by time-dependent ROC in TCGA-COAD dataset, and compared with that of gender and tumor stage. The ROC of the NTRG score remained approximately 0.9 from 500 to 4,000 days (**Figure 6H**). In the IMvigor210 dataset, 41.28% of the patients with a higher NTRG score showed sensitivity to immunotherapy. Nevertheless, the response rate declined to 18.25% when it comes to those with a lower NTRG score (**Figure 6I**). Consistently, 52.73% of patients with a higher NTRG score in the GSE173839 dataset exhibited response, whereas only 15.56% with a lower NTRG score responded to immunotherapy (**Figure 6J**).

### Landscape analysis of the NTRG score

**Figure 7:**
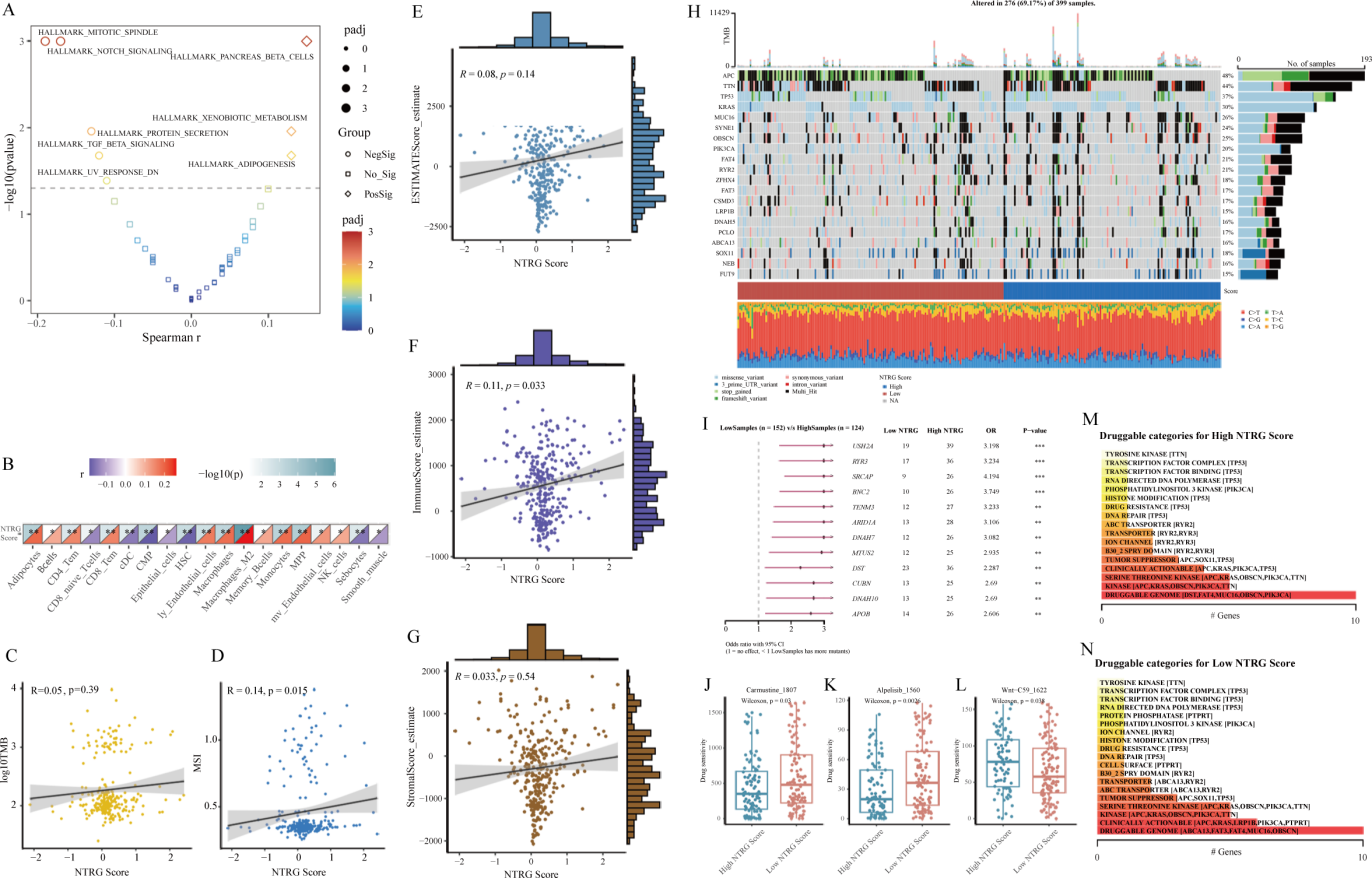
Characterization of the relationship between NTRG score and cancer microenvironment, somatic mutation, and drug prediction. **(A)** Volcano plot of the correlation between NTRG score and Hallmark cancer features. **(B)** Relationship between NTRG score and immune cell infiltration. **(C–G)** Correlation analysis between NTRG score and TMB **(C)**, MSI **(D)**, Estimate Score **(E)**, Immune Score **(F)**, and Stromal Score **(G)**. **(H)** Somatic mutation of patients with different NTRG scores. **(I)** Gene mutation difference in patients with different NTRG scores. **(J–L)** GDSC analysis of the sensitiveness of Carmustine **(J)**, Alpelisib **(K)**, and WNT-C59 **(L)** in patients with different NTRG scores. **(M, N)** Sensitive drug screening through somatic mutations in patients with higher **(M)** and lower **(N)** NTRG scores.

In order to describe the relationship between NTRG score and tumor-related biological pathways, Hallmark pathway scores were performed in TCGA-COAD followed by Spearman correlation analysis. The results indicated that HALLMARK_PANCREAS_BETA_CELLS and HALLMARK_XENOBIOTIC_METABOLISM positively correlate with the NTRG score. HALLMARK_MITOTIC_SPINDLE, and HALLMARK_NOTCH_SIGNALING pathways were negatively associated with the NTRG score (**Figure 7A**). XCell analysis was conducted to evaluate the infiltration of each type of immune cell in TCGA-COAD dataset, and Spearman correlation analysis was subsequently performed with the NTRG score. The consequence showed that the NTRG score positively contributes to NK, CD8 Tem, and macrophage M2 cell infiltration but negatively correlates with cDC, CMP, and HSC cell infiltration (**Figure 7B**). Despite of the positive correlation trend with TMB without significance, the NTRG score was testified to be positively relevant to MSI (**Figures 7C, D**). Moreover, the relevance analysis with tumor purity demonstrated that the NTRG score has a positive correlation with Immune Score but no significant relationship with Estimate or Stromal Score (**Figures 7E–G**).

Aiming for better application of NTRG scores in clinical practice, we evaluated the somatic mutation profile in populations with different NTRG scores. As is shown, somatic mutations were more frequent in patients with higher NTRG scores (**Figure 7H**). It was found that the mutations of 12 genes in patients with a higher NTRG score were more common than those with a lower NTRG score (**Figure 7I**). In addition, drug sensitivity analysis based on the GDSC drug database was performed. Patients with a lower NTRG score were more likely to be sensitive to carmustine and alpelisib, whereas those with a higher NTRG score might show response to WNT-C59 (**Figures 7J–L**). Drugs associated with somatic mutations in the higher NTRG score group covered DRUGGABLE GENOME [DST, FAT4 MUC16, OBSCN, PIK3CA] “and” KINASE/APC, KRAS, OBSCN PIK3CA, TTN]. However, agents relevant to somatic mutations in the lower NTRG score group contained DRUGGABLE GENOME [ABCA13 FAT3, FAT4, MUC16, OBSCN] “and” CLINICALLY ACTIONABLE [APC, KRAS, LRP1B PIK3CA, PTPRT] (**Figures 7M, N**).

### Expression difference analysis of NTRG in CRC tissues

**Figure 8:**
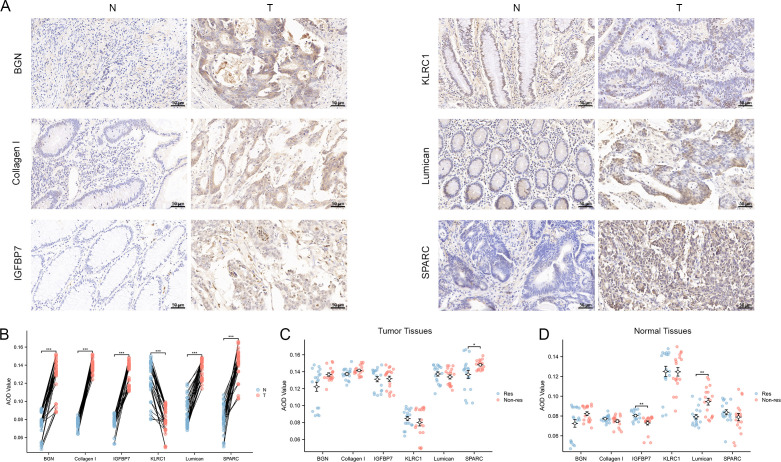
Expression of NTRG in CRC samples. **(A)** Representative IHC staining images of differential expression of NTRG (BGN, Collagen I, IGFBP7, KLRC1, Lumican, and SPARC) at the protein level in CRC and related normal tissues. **(B)** Semiquantitative analysis of the IHC staining results shown in **(A)** (n=12, *** *P*<0.001). **(C)** Expression diversity of NTRG in immunotherapy sensitive (Res, n=6) and insensitive (Non-res, n=6) CRC tissues (*P<0.05). **(D)** Expression difference of NTRG in immunotherapy sensitive (Res, n=6) and insensitive (Non-res, n=6) CRC-related normal tissues (**P<0.01).

The protein levels of NTRG were detected in six pairs of CRC and corresponding normal tissues through IHC assay. Notably, a lower expression of KLRC1 and relatively higher levels of BGN, Collagen I, IGFBP7, Lumican, and SPARC in CRC compared with paracancer tissues were proven (**Figures 8A, B**). Moreover, for the aim of exploration on the relationship between NTRG expression profiles and tumor immunoefficacy, the samples were divided into Response and Non-response groups according to the PFS. It was found in the Non-response group that SPARC expresses more abundantly in cancerous tissues (**Figure 8C**), whereas lower content of IGFBP7 and higher level of Lumican were detected in normal tissues compared with the Response group (**Figure 8D**).

## Discussion

According to the global statistics, CRC is the third most prevalent cancer worldwide and the second leading cause of cancer-related mortality, remaining to be a major public health problem and to cause severe disease burden (1). To make matters worse, the incidence of CRC is still rising dramatically in some less developed regions especially Asian and African countries (17). It is worth noting that China contributes nearly a third new cases and deaths of CRC worldwide yearly and the numbers are still growing continuously (17). It is high time that we should probe into the pathophysiological mechanism of CRC, based on which effective interventions ought to be taken to improve clinical prognosis.

As the main components of the tumor microenvironment, immune cells have been proven to be involved in the regulation of tumor malignancy (18). During the present study, we profiled a scRNA-seq dataset containing six CRC samples to explore the heterogeneity of immune cells. We detected a relatively high proportion of T cells consisting of six subgroups. Furthermore, the immune landscape and crucial cell subpopulations have been reported to impact on the molecular characteristics of CRC (19, 20). Hence, we performed pseudotime ordering to explore the potential differentiation trajectories of different T-cell subsets. Our analysis suggested that NKT cells map to an intermediate-to-late stage along the inferred trajectory, whereas Th1 cells align closely with the terminal transcriptomic state. These inferred state transitions were accompanied by sequential dynamic changes in the expression of their respective signature genes. NKT cells are unconventional T cells with both T-cell and NK cell receptors on the surface. They can play a cytotoxic role similar to NK cells via producing a large number of cytokines (21). In addition to killing CRC cells directly, the cytokines secreted by NKT cells are able to mature or activate other immune cells to exert antitumor cytotoxicity synergistically (22, 23). Furthermore, peripheral or intestinal NKT cells have been testified to exhibit suppressive efficiency against CRC through the perforin-granzyme pathway (24). Regarding clinical significance, the elevated density of NKT cells is acknowledged to be linked with longer cancer-specific survival independent of potential confounders (25). Here, through analyzing two independent data sets, we discovered that a high level of NKT cells contributes to better prognosis especially OS in CRC patients. As one of the subtypes of CD4 T cells, Th1 cells mediate cellular immunity by secreting a variety of cytokines (26). Additionally, activated NKT cells are able to reduce melanoma tumor through increasing the frequency of effector Th1 cells (27). In addition, the spatial relationship between NKT and Th1 cells is a significant predictor for pathological complete response to neoadjuvant chemoradiotherapy (28). We analyzed one spatial transcriptome sequencing sample of CRC to further probe into the potential crosstalk between different cell types. The consequences indeed indicated that NKT and Th1 cells are spatially co-localized. Given the spatial correlation, we inferred that some certain interaction may exist between NKT and Th1 cells. By collecting nine databases of CRC, we verified that there is a positive linking between the infiltration degree of these two cell types, since the cell–cell communication in the tumor microenvironment has been widely authenticated to play a critical role in the cancer progression as well as therapeutic response (29). As the results show, a number of exchange interactions were discovered between NKT and Th1 cells. Moreover, the COLLAGEN pathway was found to be the strongest signal through which NKT cells receive cellular communication. Notably, collagen rearrangement has been confirmed to participate in forming a perivascular metastatic microenvironment to facilitate CRC liver metastasis (30). We further identified that the binding of NKT cell receptor COL1A2 and the ligand CD44 on Th1 cells is able to activate the COLLAGEN signaling. Among them, a high expression of COL1A2 was closely related with shorter overall and disease-free survival in CRC with liver metastasis (31).

As one of the most commonly used immunotherapy agents, ICIs are extensively applied into treatment against multiple cancer types as its success in achieving durable responses. It is widely acknowledged that ICIs can provide significant survival improvements in CRC patients with MSI or dMMR, which constitute less than 5% of all cases (32). By contrast, the vast majority of patients featured by MSS or pMMR response to ICIs variously, which cannot be prejudged effectively by current biomarkers (33). Therefore, it is an urgent need to identify more accurate and stable biomarkers for broader clinical utility to help more CRC patients benefit from immunotherapy. Here, we firstly screened out characteristic genes of NKT and Th1 cells, among which seven vital genes correlated with prognosis and immunotherapy efficacy were further filtrated with the help of three CRC datasets. Afterward, we established an NTRG score to assess the prognostic relevance of these seven genes in CRC and their potential association with immunotherapy response. In TCGA-COAD dataset, patients with a higher NTRG score showed worse prognosis containing shortened OS, PFI, PFS, and DSS. In addition, we were surprised to discover that the ROC of the NTRG score remains at a high level even better than that of the tumor stage. In terms of the relationship with immunotherapy efficacy, we obtained a consistent result in two separate cohorts that CRC patients with a higher NTRG score show a more satisfactory therapeutic response. Here, we observed that CRC with a high NTRG score exhibits a paradoxical immune phenotype. On one hand, the active recruitment and infiltration of NKT and Th1 cells maintain an immunologically hot tumor microenvironment, which mechanistically explains the relatively favorable objective response rates to immune checkpoint inhibitors. On the other hand, a higher expression of parts of the genes among NTRG may contribute to the dense physical barrier formed by the extracellular matrix coupled with the M2 macrophage-mediated immunosuppressive niche which severely impairs the antitumor efficacy of these infiltrating effector cells. Consequently, the inability to effectively eradicate tumor cells ultimately translates into worsened prognosis. The coexistence of a desmoplastic stromal signature with pro-immunogenic NKT/Th1 activity reflects a dynamic immune-ecological tension inherent to the CRC microenvironment. Rather than representing mutually exclusive states, cold stromal enrichment and hot immune engagement are functionally coupled. The desmoplastic matrix acts as both a physical barrier excluding effector lymphocytes and a chemotactic scaffold that recruits and spatially organizes innate-adaptive immune cells, including NKT and Th1 populations. In this context, elevated NTRG expression may mark a transitional microenvironmental state in which immune surveillance is actively engaged but cytotoxic execution is mechanically restrained by matrix-mediated exclusion. This model aligns with the concept of immune-excluded tumors, which harbor substantial lymphocyte infiltration at stromal boundaries yet fail to achieve intratumoral penetration. The NTRG score therefore captures not a unidirectional cold or hot phenotype but the degree of stromal-immune interface tension, which may determine whether immunotherapy can convert a constrained immune response into productive tumor clearance. The MSI and TMB status has been generally acknowledged to lead to immunological heterogeneity and routinely incorporated into clinical practice to guide immunotherapy decisions (34, 35). In our present research, we detected that the NTRG score displays a close relationship to kinds of pathways associated with tumor metabolism and carcinogenicity. Beyond that, a higher NTRG score was found to positively contribute to both MSI and TMB values. As is broadly regarded, it is the diversity of tumor-infiltrating immune cells that results in disparate outcomes of patients responding to immunotherapy (36). We testified that the NTRG score is positively linked with NK, CD8 Tem, and macrophage M2 cell infiltration but negatively gives rise to that of cells such as cDC, CMP, and HSC. In addition, a correlation of statistical significance between NTRG score and Immune Score was further confirmed. Although most mutations in somatic cells do not have a noticeable effect, progressive accumulation of mutations in those crucial driver genes has been verified to trigger malignancies (37). From the consequence of our somatic mutation landscape analysis, it could be demonstrated that patients with higher NTRG scores possess more frequent genetic mutations, suggesting a more active mutation spectrum potentially associated with tumor immunogenicity. It is commonly recognized that somatic mutations in cancer genomes are essential for targeted therapeutics development (38). In view of drug sensitivity on the basis of the GDSC drug database, we probed a series of genes with potential druggability on account of the somatic mutations in both groups. In addition, we speculated that patients with a higher NTRG score might be sensitive to WNT-C59-related agents, whereas those with a lower NTRG score could show better response to carmustine or alpelisib. Aiming for deeper clinical significance, expression profiles of NTRG were researched in paired CRC samples. Reduced content of KLRC1 and increased expression of BGN, Collagen I, IGFBP7, Lumican, and SPARC were discovered in tumors compared with normal tissues. Furthermore, compared with the Response group, SPARC was found to express more highly in cancerous tissues, whereas lower content of IGFBP7 and higher level of Lumican were detected in normal tissues of the Non-response group. Therefore, we believe that the unique value of the NTRG score as a composite biomarker lies in its integration of dual signals from both the immune and stromal components and that it is the heterogeneity of the expression of these molecules in tumor tissues as well as its microenvironment that leads to the diversity of the therapeutic efficacy of immunotherapy.

## Conclusions

In brief, our present research probed into the characteristics of the immune microenvironment of CRC from the perspective of single-cell and spatial transcriptomics. More importantly, we used NKT-Th1-cell-related genes to construct an NTRG score, which may help characterize the immune landscape of CRC and its associations with immunotherapy response and prognosis. We firmly believe that prospective validation ought to be performed based on the present results to benefit more CRC patients.

## Limitations

Although underlying valuable findings, the current study has a few limitations, including inherent bias risk of the retrospective research design, heterogeneity of clinical information integrity in public databases, and technical challenges faced when converting the score based on transcriptome characteristics into the routine clinical pathological detection workflow. Future prospective cohort studies should systematically collect comprehensive clinical pathological parameters, including the metastasis sites and treatment history, to verify the independent predictive value of the NTRG score in a more homogeneous population. In addition, it is worth conducting head-to-head comparisons and evaluations of the combined application value of the NTRG score with indicators such as MSI and TNM stage to more comprehensively define the clinical application scenarios. Verification of the predictive efficacy of the NTRG score in the MSS population ought to be conducted for the incremental value of NTRG in the precise prognostic management of CRC. Additionally, reaction evaluation standards specifically for immunotherapy, such as irRECIST or iRECIST, and the time point of confirmation scans should be adopted to obtain more accurate efficacy assessment. Last but not least, a detailed subclustering analysis in a larger sample size of single-cell data, together with *in vitro* and *in vivo* function verification need to be conducted to explore the regulatory mechanism between NKT and Th1 cells and potential functional subgroups.

## Data Availability

The original contributions presented in the study are included in the article/**Supplementary Material**. Further inquiries can be directed to the corresponding authors.
