# Supplementary figures and images for: Integrated single-cell and spatial transcriptomics reveal immune landscape and NKT–Th1 signatures in colorectal cancer
Source: Front Immunol. 2026 Jun 10;17:1774363. doi: 10.3389/fimmu.2026.1774363 (PMC13291139; doi:10.3389/fimmu.2026.1774363)

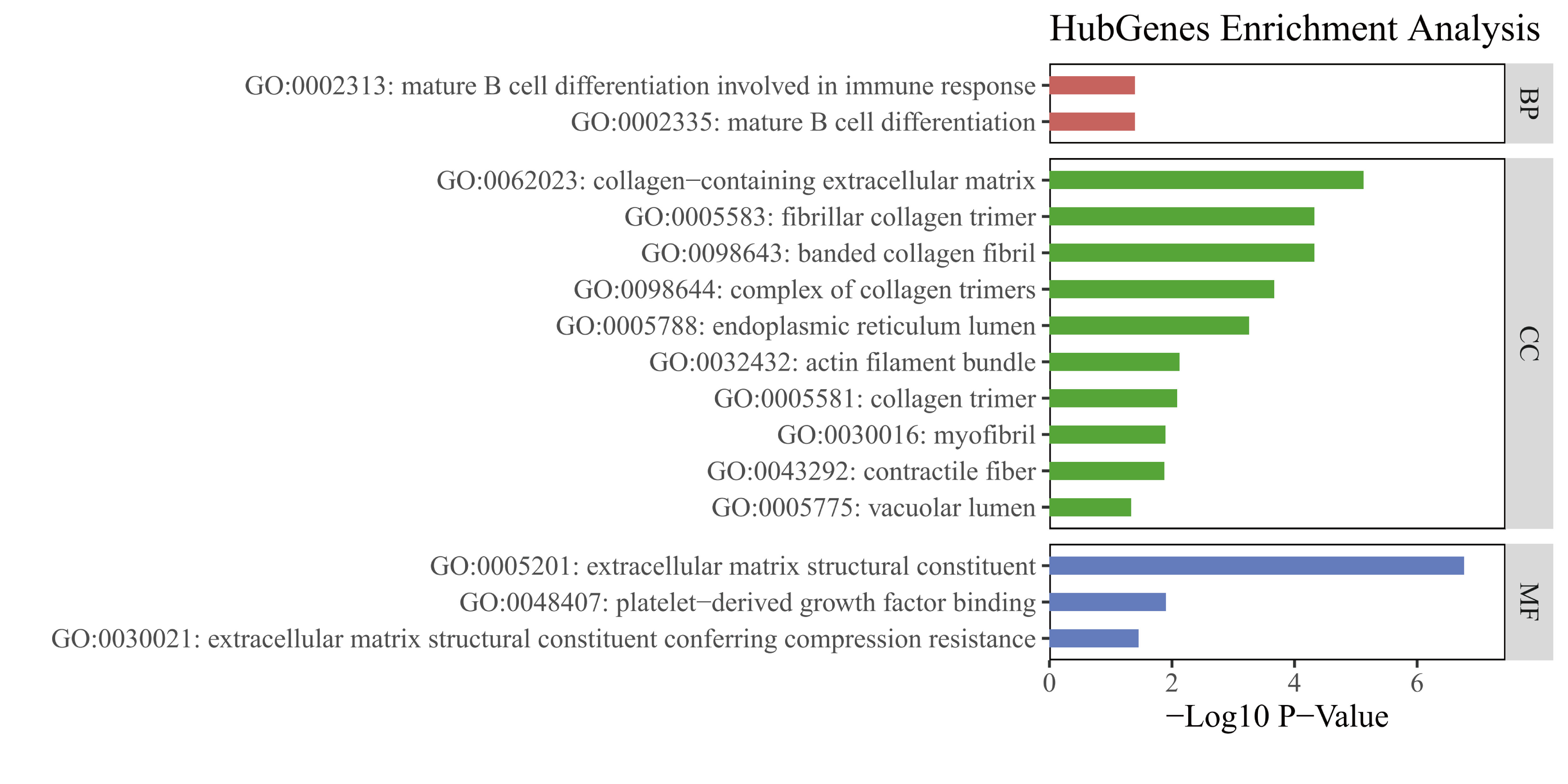

Supplement: Supplementary file 1 [file Image1.tif]
