# Supplementary material for: Integrated single-cell and spatial transcriptomics reveal immune landscape and NKT–Th1 signatures in colorectal cancer
Source: Front Immunol. 2026 Jun 10;17:1774363. doi: 10.3389/fimmu.2026.1774363 (PMC13291139; doi:10.3389/fimmu.2026.1774363)
Supplement: Supplementary file 4 [file Table3.docx]

| **TITAN Guideline Checklist 2025** | | | |
| --- | --- | --- | --- |
| **Topic** | **Item** | **Description** | **Page**  **number** |
| **Artificial**  **Intelligence (AI)**  **(some journals may prefer this in the**  **methods and/or**  **acknowledgments section and it**  **should also be declared in the cover letter)** | 5 | **Declaration of whether any AI was used in the research and manuscript development**  **If no, proceed to item 6.**  **If yes, proceed to item 5a** | NO |
|  | 5a | **Purpose and Scope of AI Use**  - Precisely state why AI was employed (e.g. development of research questions, language drafting, statistical  analysis/summarisation, image annotation, etc).  - Was generative AI utilised and if so, how?  - Clarify the stage(s) of the reporting workflow affected (planning, writing, revisions, figure creation).  - Confirmation that the author(s) take responsibility for the integrity of the content affected/generated |  |
|  | 5b | **AI Tool(s) and Configuration**  - Name each system (vendor, model, major version/date).  - State the date it was used  - Specify relevant parameters (e.g. prompt length, plug-ins, fine- tuning, temperature).  - Declare whether the tool operated locally on-premises, or via a cloud API and any integrations with other systems. |  |
|  | 5c | **Data Inputs and Safeguards**  - Describe categories of data provided to the AI (patient text, de- identified images, literature abstracts).  - Confirm that all inputs were de-identified and compliant with GDPR/HIPAA.  - Note any institutional approvals or data-sharing agreements obtained. |  |
|  | 5d | **Human Oversight and Verification**  - Identify the supervising author(s) who reviewed every AI output.  - Detail the process for fact-checking, clinical accuracy checks  - State whether any AI-generated text/figures were edited or discarded.  - Acknowledge the limitations of AI and its use |  |
|  | 5e | **Bias, Ethics and Regulatory Compliance**  - Outline steps taken to detect and mitigate algorithmic bias (e.g. cross-checking against under-represented populations). |  |

|  |  | - Affirm adherence to relevant ethical frameworks.  - Disclose any conflicts of interest or financial ties to AI vendors. |  |
| --- | --- | --- | --- |
|  | 5f | **Reproducibility and Transparency**  - Provide the exact prompts or code snippets (as supplementary material if lengthy).  - Supply version-controlled logs or model cards where possible.  - if applicable, state repository, hyperlink or digital object  identifier (DOI) where AI-generated artefacts can be accessed, enabling attempts at independent replication of the query/input. |  |
